# Identification of biomarkers for bull fertility using functional genomics

**DOI:** 10.1590/1984-3143-AR2022-0004

**Published:** 2022-05-02

**Authors:** Muhammet Rasit Ugur, Denise D. Guerreiro, Arlindo A. Moura, Erdogan Memili

**Affiliations:** 1 IVF Michigan, Bloomfield Hills, MI, USA; 2 Departamento de Zootecnia, Universidade Federal do Ceará, Fortaleza, CE, Brasil; 3 Núcleo de Pesquisa e Desenvolvimento de Medicamentos, Faculdade de Medicina, Universidade Federal do Ceará, Fortaleza, CE, Brasil; 4 Department of Animal and Dairy Sciences, Mississippi State University, Starkville, MS, USA; 5 Cooperative Agricultural Research Center, Prairie View A&M University, Prairie View, TX, USA

**Keywords:** bull sperm, bull fertility, fertility biomarkers, functional genomics

## Abstract

Prediction of bull fertility is critical for the sustainability of both dairy and beef cattle production. Even though bulls produce ample amounts of sperm with normal parameters, some bulls may still suffer from subpar fertility. This causes major economic losses in the cattle industry because using artificial insemination, semen from one single bull can be used to inseminate hundreds of thousands of cows. Although there are several traditional methods to estimate bull fertility, such methods are not sufficient to explain and accurately predict the subfertility of individual bulls. Since fertility is a complex trait influenced by a number of factors including genetics, epigenetics, and environment, there is an urgent need for a comprehensive methodological approach to clarify uncertainty in male subfertility. The present review focuses on molecular and functional signatures of bull sperm associated with fertility. Potential roles of functional genomics (proteome, small noncoding RNAs, lipidome, metabolome) on determining male fertility and its potential as a fertility biomarker are discussed. This review provides a better understanding of the molecular signatures of viable and fertile sperm cells and their potential to be used as fertility biomarkers. This information will help uncover the underlying reasons for idiopathic subfertility.

## Introduction

There is an urgent need to increase the efficiency and sustainability of animal food production in the face of an ever-increasing world population (United Nations, 2017). With limitations on natural sources in the world, the need for increasing efficiency, productivity, and sustainability of the food production methods becomes highly important to meet ever-increasing food demand. Reproductive inefficiency is one of the limiting factors for the beef cattle industry because the profitability of a cow-calf operation is linked to the maintenance of the lifetime reproductive status (Moorey and Biase, 2020). Reproductive losses cause approximately $1 billion economic loss in cattle industry each year in the US (Bellows et al., 2002) and approximately 33% of the cows are removed from the beef herd due to reproductive failures (USDA, 2010).

Fertility, the competency of sperm to fertilize and activate the egg to maintain embryo development, has great economic impact on agri-food industry (Abdollahi-Arpanahi et al., 2017). Fertility is influenced by a number of factors including genetics, epigenetics, environment, and management (Parisi et al., 2014). Agriculture industry has been paying a great attention to optimize environmental and management conditions for female fertility through the reproductive biotechnologies (artificial insemination, *in vitro* embryo production and embryo transfer), estrus synchronization protocols, and various feed additives (Butler et al., 2019). Despite the increased adoption of estrus synchronization and artificial insemination (AI) in the beef cattle industry, the prevalence of these biotechnologies in the meat industry is still extremely low, especially when compared to the dairy industry, which reports 89.3% of all operations using some methods of artificial insemination (USDA, 2018). A limiting factor for the adoption of AI for most beef cattle producers, besides labor and costs, is the low likelihood of pregnancy at the first insemination (Moorey and Biase, 2020).

Bull fertility is important for the overall cattle operation because a single ejaculate from a bull distributed by breeding companies can be used to inseminate thousands of cows around the world (Braundmeier and Miller, 2001). Although bulls produce large amounts of sperm with normal morphology and motility, some animals may still suffer from infertility or subfertility (Memili et al., 2012). These substantial differences in fertility between individual bulls cause large reproductive losses since semen one bull can be used to inseminate hundreds of thousands of cows (Kastelic, 2013). Therefore, exploring critical aspects of male fertility will shed light on the infertility secrets and can have positive impacts on the AI protocols.

Many studies have been conducted over recent decades to uncover the effects of semen quality parameters on sire fertility. The need to examine fertility of the bull in North America emerged in 1949 to identify consequences of snowstorm happened in the Rocky Mountain states (Hopper, 2014). The motility of the semen samples obtained from these bulls was evaluated and this activity is assumed to be the first actual practice of fertility evaluation in the US (Hopper, 2014). The early standards for evaluation of bull fertility were set by a society known as “Rocky Mountain Society for the Study of Breeding Soundness,” and this organization is currently known as “Society for Theriogenology” (SFT) (Hopper and King, 2014).

Breeding soundness examination (BSE) is a systematic method to assess the reproductive potential of a given bull, and current standards of BSE were adopted by STF (Chenoweth et al., 2010). According to the BSE requirements, a bull is evaluated in four categories including physical examination, scrotal circumference, sperm progressive motility and sperm morphology. A bull should be able to pass all these four evaluation criteria to be considered as a satisfactory breeder. Even though BSE provides basic information about bull fertility, there are some potential limitations that may mislead the results. For instance, the current format of BSE does not evaluate the libido nor takes into account molecular aspects of sperm cells such as their DNA and membrane integrity.

There is ongoing research focused on the identification of new markers to estimate bull fertility. Evaluation of arterial blood flow to the testes through an ultrasonography is one of the potential methods to contribute to proper assessment of male fertility (Velasco and Ruiz, 2020). Batissaco et al. (2014) reported an association between Resistive Index (RI) and Pulsatility Index (PI) parameters and total sperm defects in rams, and Ortiz-Rodriguez et al. (2017) claimed that RI and PI values at the marginal testicular artery (MTA) were greater in infertile than in fertile stallions. In addition, RI and PI at the supratesticular artery (STA) were associated with sperm membrane integrity and End Diastolic Volume (EDV) at the STA (Lemos et al., 2020). Moreover, the total sperm concentration of donkeys was negatively correlated with EDV, Peak Systolic Volume (PSV), and Time-Averaged Mean Velocity (TAMV) (Gacem et al., 2020). Correlations among sperm concentration, teratoid sperm and immature concentration with RI at the MA and IT have been reported in the bull as well (Gloria et al., 2018).

Based on the current literature, it becomes clear that scientists have significant knowledge about male reproductive physiology but there is no single method or parameter that can precisely estimate bull fertility. This scenario indicates, therefore, that novel fertility markers are required, as well as more comprehensive methodologies and updated techniques for assessment of sperm quality and viability. Novel biomarkers (proteins, small noncoding RNAs, lipids, metabolites or epigenomic markers) coupled with computational analyses can be used as integrated approaches to better understand spermatogenesis and sperm quality, and to predict male fertility. High throughput screening methods combined with traditional protocols could be a better approach to understand molecular, cellular, and physiological underpinnings of bull fertility. Better understanding of sperm molecular and functional signature associated with bull fertility using systems biology approach along with systems physiology concepts is expected to enhance both the fundamental science of reproduction and assisted reproductive technologies (ART).

## Sperm signatures

### Sperm proteome and fertility

**Figure 1:**
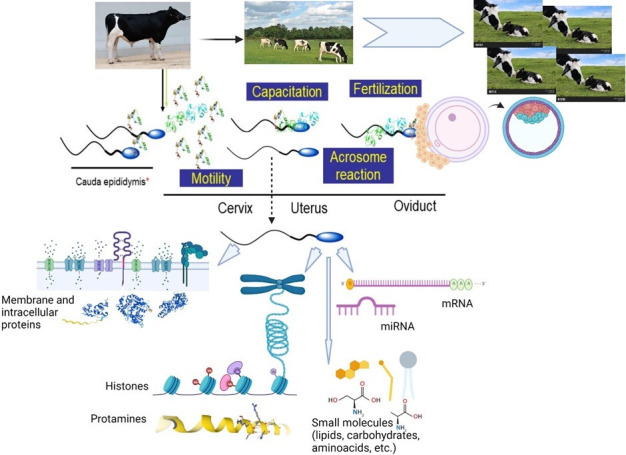
Overview of the major trajectory of sperm cells in a mammalian model. During ejaculation, cauda epididymal sperm are bathed by seminal plasma components and released into the female reproductive tract. Also, factors that are intrinsic to the male gamete such as intracellular and membrane proteins, metabolites, RNA species and the organization of its chromatin play vital roles in the process of fertilization and early embryo development. Main template of the **figure was** built using BioRender (2022) platform and structure of some proteins were downloaded from AlphaFold Protein Structure Database (AlphaFold, 2022).

Sperm are formed in the seminiferous tubules after a complex series of mitotic and reductional divisions, starting from a reservoir of spermatogonium stem cells. In the process of spermatozoa formation, the structure of sperm chromatin changes in such a way that most histones are replaced by protamines, and sperm are thought to become transcriptionally inactive (Grunewald et al., 2005; Miller et al., 1999; Miller and Ostermeier, 2006; Ren et al., 2017; Selvaraju et al., 2018a). This alleged halt in gene transcription capacity is believed to be already present in sperm cells released into the lumen of the seminiferous tubules. However, the “dogma” of sperm´s inability to make proteins has been the subject of debate. Sperm cells may not have all the RNA polymerase and the transcription machinery to fully transcribe genes like active diploid cells. However, pieces of experimental evidence indicate that sperm may have some capacity of making proteins from RNAs already present in the cells after the completion of spermatogenesis (Dai et al., 2019). Alternatively, sperm cells, at some point during their development, maturation and beyond may have some transcription activity as well (Giordano et al., 2000; Ren et al., 2017; Wang et al., 2018). From the rete testis, immature sperm enter the convoluted epididymal pipes, undergo maturation and are further mixed with secretions of the accessory sex glands. Sperm acquire proteins from the milieu where they are maintained in the epididymis. Thus, the protein composition of spermatozoa comprises intracellular proteins, membrane proteins of the cell, and proteins attached to them coming from the epididymal and accessory sex gland fluid (Kenny and Byrne, 2018; Rego et al., 2016; van Tilburg et al., 2021). The major trajectory and functional events of sperm cells in a mammalian model are summarized in Figure 1.

Researchers have dedicated significant efforts to the task of mapping the complete proteome of sperm cells. Proteomic studies are more advanced in humans, where nearly 6,200 sperm proteins have been described so far, from the universe of estimated 7,500 proteins (Codina et al., 2015). It is difficult to project how many proteins ejaculated bull sperm have but it is plausible to assume that the total number of proteins present in the male gamete is similar across mammalian species. In addition to the number of sperm proteins, our understanding about the types and functions of those proteins becomes crucial as well. Also, clarifying how specific proteins are related to the physiology and kinetics of sperm is critically important to define if any sperm proteins are the true markers of male fertility in the bovine species. Based on studies conducted in humans, sperm proteins are diverse in structure and function, acting as contractile components and to regulate the motility, phosphorylation, energy metabolism and chromatin organization. Sperm also contain a complex array of membrane proteins, many of which modulate ion channels, sperm binding to the epithelial cells of the female reproductive tract, acrosome reaction, sperm-egg recognition and fertilization (Selvaraju et al., 2018b). In humans, functional pathways associated with sperm proteins have been mainly defined as metabolism, protein metabolism, membrane trafficking, RNA metabolism, apoptosis, cell cycle, hemostasis, and meiosis (Amaral et al., 2013). All these characteristics are not surprising because authors have postulated that specific proteins are related to cases of infertility or subfertility in men (Liang et al., 2021).

For the bovine species, coverage of the global proteome of sperm has reached significant milestones but it is still limited, with almost 3,000 proteins being identified so far (Byrne et al., 2012; Laxmivandana et al., 2021; Ramesha et al., 2020). As mentioned in reproductive studies, analyses of bull sperm have indicated empirical relationships among sperm proteins, parameters of both fresh and frozen-thawed sperm, and fertility indexes (D’Amours et al., 2019; Gaviraghi et al., 2010; Peddinti et al., 2008; Rego et al., 2014; Westfalewicz et al., 2021). Recent research results show that the proteome of sexed and non-sexed bovine sperm are distinct and that Y- and X-chromosome bearing sperm have different protein signatures (Mostek et al., 2020). Taken together, these pieces of information support the concept that sperm quality and even the actual fertility of the bulls can be potentially tracked back to the proteome of the sperm cells. In the bull sperm, Protamine 1 (PRM1), and postacrosomal assembly of sperm head protein (PAWP) and outer dense fiber of sperm tails 2 (ODF2) have been identified as potential fertility markers by Dogan et al. (2015) and Kaya et al. (2022).

Most studies present large variations due to various methods used to extract and analyze the proteins, breed, and sub-species of bulls (*Bos indicus vs. Bos taurus*) and as such there is a need for discovering definitive biomarkers of bull fertility or sperm freezability based on sperm proteins. Such variations in studies about biomarkers of bull fertility have been also noticed when fertility-associated proteins of the seminal plasma were recently analyzed in a comprehensive review (Viana et al., 2021). In the future, larger studies and standardized proteomic approaches will probably help to decipher the reliable molecular indicators of sperm quality and bull fertility. These biomarkers could then be used to help the selection of superior sires.

Modern, state-of-the-art pieces of equipment and bioinformatics tools allowed researchers to uncover differences in protein abundances in the sperm from animals, foreseeing the definition of reliable markers of sire reproductive traits. Most studies cited above, if not all, used classical, bottom-up proteomics to identify and quantify sperm proteins from animals with contrasting fertility and sperm parameters. Bottom-up approaches for proteomics have brought exceptional knowledge about the protein composition of sperm but some information was lost with that technical strategy. Top-down proteomics, instead, analyzes intact proteins and allows identification of protein complexes, isoforms and mapping of post-translational modifications (PTMs) (Gomes et al., 2019; Toby et al., 2016). Protein isoforms and PTMs define important pathways showing how certain proteins function in biological systems, including the seminal plasma (Janiszewska and Kratz, 2020; Singh et al., 2019) and sperm (Baker, 2016; Soler-Ventura et al., 2020). Thus, deep analyses of sperm proteins using top-down strategies should be one of the future directions for male gamete research. Another relevant aspect of studies focused on sperm proteins is the fact that the population of sperm cells in a single ejaculate is not homogeneous (Holt and van Look, 2004). Each sperm cell may differ in size, morphology, kinetics, metabolism, and protein composition, among other criteria, and these various characteristics affect how sperm protein markers of fertility are scrutinized. Thus, scientists should take the heterogenic sperm population into consideration in their analyses and could use single cell proteomics (SCeProt) to better understand roles of sperm proteins and their regulatory attributes to sperm physiology. This way, specific protein trademarks can be linked to sperm motility, survival, and fertilizing capacity in an accurate way for the identification of bull fertility.

### Sperm small noncoding transcripts and fertility

Over many years, scientists supposed that the great majority of the genome that is noncoding part of DNA was “junk” because no noticeable function had been identified for this portion (Watson et al., 2019). In recent years, however, it has been identified that approximately 20,000 protein-coding genes are actually regulated by such noncoding regions (Wright and Bruford, 2011). After the realization of the biological roles of non-coding genes, the discovery of non-coding RNAs (ncRNA) in biomarker studies accelerated as well (Watson et al., 2019). Small noncoding RNAs (sncRNA) are short non-coding sequences, 18 to 200 nt long, and modulate a number of biological processes such as posttranscriptional regulations, ribosome biosynthesis and gene silencing (O’Brien et al., 2018; Reichow et al., 2007). A number of sncRNA classes have been reported, including microRNA (miRNA), small interfering RNAs (siRNA), small nucleolar RNAs (snoRNA), small nuclear RNA (snRNA), PIWI-interacting RNA (piRNA). Even though sperm are considered, in some extent, transcriptionally inactive, increasing evidence demonstrates that mature sperm carry several long-coding RNAs and sncRNA (Erickson, 1990; Vibranovski et al., 2010).

While there is no common consensus about the origin of RNAs in sperm, it is generally accepted that RNAs are left over molecules from spermatogenesis (Dadoune et al., 2004; Hilz et al., 2016). The exact role of sperm borne sncRNAs are not well understood. However, recent evidence suggests that sperm borne miRNAs are transferred into oocytes during fertilization (Reilly et al., 2016), and potentially play a role in early embryonic development (Alves et al., 2019). Similarly, potential functions of paternal miRNAs and endo-siRNAs in fertilization, zygote and two-cell embryo development were reported in mouse (Yuan et al., 2016). A total of 959 miRNA candidates were detected in bovine sperm using high throughput sequencing technology (Du et al., 2014). Also, differences exist in the miRNA profiles of sperm from high and low fertility bulls (Govindaraju et al., 2012), and differential expressions of seven sperm-borne miRNAs (mir-502-5p, mir-1249, mir-320a, mir-34c-3p, mir-19b-3p, mir-27a-5p and mir-148b-3p) have been reported for bulls with moderate and high non-return rate (Fagerlind et al., 2015). More recently, higher expression of miR-15a and miR-29 in low fertility compared to high fertility bulls were reported as well (Menezes et al., 2019). To this end, individual or global expression of sncRNAs could be considered as potential fertility biomarkers for the diagnosis of male infertility.

### Sperm lipidome and fertility

Lipids are building blocks of cell membranes and consist of a large group of heterogeneous chemical compounds with varying structure and biological functions (Nigam and Singh, 2014; Santos and Preta, 2018). Numerous studies have demonstrated that the composition of lipids plays an important role in signaling, membrane trafficking, energy metabolism and cellular metabolism (Carvalho et al., 2018; Klose et al., 2013; Muro et al., 2014; Wymann and Schneiter, 2008). The membrane of the sperm is critical for fertilization because of its role in spermatozoon-oocyte cross-talk and membrane fluidity (Lenzi et al., 1996). The most abundant component of the sperm membrane is phospholipids (70%), flowed by neutral lipids (25%), and glycolipids (5%) (Rivera-Egea et al., 2018).

The bilayer of the sperm plasma membrane is predominantly made of phospholipids and fatty acids, and saturation levels of fatty acids could be a potential indicator for sperm parameters (Lenzi et al., 1996). Polyunsaturated fatty acids (PUFAs) are prone to lipid peroxidation as opposed to saturated fatty acids (SFAs) and monounsaturated fatty acids (MUFAs) (delBarco-Trillo and Roldan, 2014; Hulbert et al., 2014). A high abundance of PUFA makes sperm more vulnerable to lipid peroxidation caused by reactive oxygen species (ROS) and may impair sperm functional parameters (Aitken, 1990; Aitken et al., 1989; Alvarez and Storey, 1982). In addition, Hossain et al. (2007) determined the effects of fatty acids on boar sperm parameters including motility, viability, and acrosome reaction. Their results revealed that supplementation of sperm with oleic and linoleic acid improved motility and viability rates while oleic and arachidonic acid enhanced acrosome reaction. delBarco-Trillo et al. (2013) conducted a comparative study among mammals to investigate the role of fatty acid composition on sperm competitions and discovered that a higher level of sperm competition correlated with a decreased proportion of the sperm PUFAs. Evans et al. (2020), in turn, demonstrated that Arachidic acid (20:0), Oleic acid (18:1 cis 9), Myristic acid (14:0 13-methyl fatty acids) have differential abundances in sperm of bulls with contrasting freezability phenotypes. More recently, it has been reported that supplementation of frozen-thawed bull sperm with saturated long chain fatty acids, including myristic acid, palmitic acid, margaric acid and stearic acid, increased linear motility of sperm (Islam et al., 2021). Thus, the composition of lipid fractions in the membrane could be a potential determinant of sperm fertilization ability.

### Sperm metabolome and fertility

Metabolomics is an emerging technology for the discovery of biomarkers and provides a snapshot of biological processes through the measurement of metabolites and investigation of metabolomic pathways (Powers and Riekeberg, 2017). Since metabolites are the end products or intermediates of metabolic reactions, they could provide a broad understanding of phenotypic traits (Fiehn et al., 2000). Over the last few years, metabolomics approaches gained great attention to uncover molecular, cellular, and physiological underpinnings of fertility (Egea et al., 2014).

Initial metabolomics studies in reproductive studies were focused on the detection of oxidative stress markers (-CH, -NH, -OH, -SH) in human sperm, oocyte, and embryo (Deepinder et al., 2007). More recent metabolomic studies in animal reproduction aimed at profiling seminal plasma and sperm cell composition to detect both multivariate and single biomarkers. Kumar et al. (2015) profiled seminal plasma from high fertility bulls using proton nuclear magnetic resonance (^1^H NMR) and revealed that taurine, isoleucine, and leucine of seminal plasma are potential biomarkers of bull fertility. Similarly, Velho et al. (2018) analyzed the seminal plasma from high fertility and low fertility Holstein bulls using gas chromatography-mass spectrometry (GC-MS). These authors identified a total of 63 metabolites in seminal plasma samples of Holstein bulls, and demonstrated that fructose, citric acid, lactic acid, urea, and phosphoric acid were the most abundant ones. Univariate analysis of metabolomic data showed that the abundance ration of 2-oxoglutaric acid, ornithine, L-leucine, and D-mannitol were greater in low fertility bulls than those in high fertility bulls. In addition, Menezes et al. (2019) reported that abundance ratios of gamma-aminobutyric acid (GABA), carbamate, benzoic acid, lactic acid, and palmitic acid were different in sperm of high fertility and low fertility bulls. More recently, Ugur et al. (2020) showed that abundance of phenylalanine in seminal plasma is a potential biomarker of Holstein bull sperm freezability due to its antioxidant effect. Lastly, the effects of cryopreservation on the metabolic profile of sperm and seminal plasma from high and low fertility bulls were evaluated using liquid chromatography-mass spectrometry (LC-MS) by Longobardi et al. (2020). According to this study, cryopreservation caused an increase in the content of lysophosphatidylcholine in seminal plasma, and that of glycine betaine and pyro-l-glutaminyl-l-glutamine in sperm. Based on the same study, fresh seminal plasma from high fertility bulls had more L-acetylcarnitine, glycerol tripropanoate, 2,3-diacetoxypropyl stearate and glycerophosphocholine while levels of lysophosphatidylcholine and butyrylcarnitine were lower in the high fertility bulls.

### Sperm chromatin dynamics and fertility

The chromatin structure of spermatogonia consists of nucleosomes and each nucleosome is made of DNA wrapped around nuclear histones comprised of H2A, H2B, H3, and H4 (Talbert and Henikoff, 2010). Such histones are small-sized nuclear proteins that are positively charged due to arginine and lysine residues in their structures (Andrews and Luger, 2011; Richmond and Davey, 2003). The positive charge facilitates a strong link between histones and negatively charged DNA, which is critical for a compact chromatin at the early stages of spermatocytogenesis (Bao and Bedford, 2016). In addition to the core histones, H1 protein interacts with the linker DNA region, helping better compaction of the chromatin by stabilizing chromatin fibers (Drabent et al., 1997; Ward, 2010).

During the meiotic and post-meiotic stages of spermatogenesis, spermatids undergo a chromatin remodeling process to protect the paternal genome (Bao and Bedford, 2016). This remodeling process is accompanied by PTMs of histones with the incorporation of testis-specific histone variants (Bao and Bedford, 2016; Champroux et al., 2016; Hao et al., 2019). Initially, hyperacetylation of the histones reduces DNA/histone interaction, and bromodomain testis-specific protein (Brdt) binds to these hyperacetylated histones (Oliva et al., 1987; Oliva and Mezquita, 1986). Loosened chromatin allows the replacement of histones with transition proteins (TP) and it is followed by the exchange of TP with PRMs (Balhorn, 1982; Eirín-López et al., 2006; Ward, 2010). Sperm DNA-protamine interaction is provided by inter- and intra-protamine disulfide bonds, and mature sperm chromatin has a toroidal structure that contains 50 to 100 kb of DNA coiling around protamine toroid subunits (Brewer et al., 2003; Hud et al., 1993). Such process makes the sperm cell DNA more condensed than somatic cells, which is critical for fertilization because sperm cells are exposed to chemical and physical damage moving through the female reproductive tract (Champroux et al., 2016).

Post-translational modifications of histones include acetylation, methylation, phosphorylation and ubiquitylation (Allfrey et al., 1964; Walsh et al., 2005). Such modifications can impact gene expression by modulating chromatin structure and initiating chromatin remodeling and reorganization (Bannister and Kouzarides, 2011; Spencer and Davie, 1999). Acetylation reduces the affinity of histones for DNA by neutralizing the positive charge of lysine residues on the amino-terminal tails of histones (Walsh et al., 2005). Among those PTMs, H4 acetylation modulates different stages of spermatogenesis. For example, hyperacetylation of H4 (Hypac-H4) was detected in spermatogonia and spermatids, while acetylated lysine-12 of histone-H4 (Lys12ac-H4) was detected only in the mouse spermatids (Hazzouri et al., 2000). In addition, PTM of H4 modulates histone replacement by affecting protein-protein interactions by apoptotic nuclease DNA fragmentation (Bošković and Torres-Padilla, 2013; Hao et al., 2019; Polo and Jackson, 2011). It has been reported that double bromodomain-containing binds to acetylated H4 and thus causes more relaxed chromatin for histone removal, consequently facilitating condensation of the nucleus (Maeshima et al., 2010).

It has been suggested that insufficient histone replacement causes abnormal elongation (Cho et al., 2003) and inefficient chromatin decondensation in mammalian sperm (Kazerooni et al., 2009). The proposed mechanisms assume that retained histones make sperm chromatin less condensed, and therefore loose paternal genome becomes more vulnerable to mutagenesis and damage (Bao and Bedford, 2016). Sperm head shape and size affect velocity and hydrodynamics of the sperm cell (Tourmente et al., 2011): A more elongated and condensed sperm head makes sperm cells more motile and protects the paternal genome from chemical damage in the female reproductive tract (Rathke et al., 2014). The abundance of both retained histones and protamines is critical for sperm physiology, chromatin integrity and embryo development because of their indispensable role in sperm maturation (Franken et al., 1999; Zhang et al., 2016). De Oliveira et al. (2013) compared relative quantities of H2B, H3.3, and H4 in mature sperm from high fertility and low fertility bulls, and no differences were detected between these two groups of sires. The functions of H3.3 in mice fertility and gametogenesis were evaluated by Tang et al. (2015) through the analyses of null mutations of *H3f3a* and *H3f3b* genes. Their findings revealed that H3.3 is a potential marker for gametogenesis and fertilization. An inverse correlation was also reported between expression levels of testis-specific histone 2B (TH2B) and bull fertility (Kutchy et al., 2017).

Several studies reported that the ratio of protamine 1 (PRM1) to protamine 2 (PRM2) in mature sperm is associated with DNA fragmentation, sperm motility, and sperm morphology (Aoki et al., 2005; Delbes et al., 2007; Hammadeh et al., 2010; Mengual et al., 2003; Nasr-Esfahani et al., 2004; Simon et al., 2011). In addition, the relative abundance of PRM1 and PRM2 affects the outcomes of assisted reproductive techniques (Aoki et al., 2006; 2005; Simon et al., 2011). Abnormal amounts and localization of PRM1 were associated with defects in sperm chromatin structure and reduced fertility in bulls (Dogan et al., 2015). The potential roles of acetylated H3 lysine 27 (H3K27ac), acetyl-Histone 4 (H4 acetyl) and methylated H3 (H3K27me3) were analyzed by Kutchy et al. (2018) and Ugur et al. (2019), indicating that bull fertility is modulated by H3K27me3 and H4 acetyl.

## Conclusions

The technological advances achieved in the last decades in the areas of proteomics, metabolomics, transcriptome, and genomics, have allowed the identification of possible biomarkers related to bull fertility. All these have contributed to drawing a profile, or signatures of bull sperm associated with fertility that is of extreme importance to the cattle industry since many losses are related to low rates of return after insemination of cows. However, there is still a great divergence in the results obtained, mainly due to the methodology employed and the differences between Bos Taurus and Bos indicus. Therefore, high resolution mapping of semen from bulls with reliable fertility phenotypes using advanced techniques is needed for the discovery of sperm biomarkers of fertility for precision livestock reproduction and agriculture.
